# High Precision Positioning with Multi-Camera Setups: Adaptive Kalman Fusion Algorithm for Fiducial Markers

**DOI:** 10.3390/s20092746

**Published:** 2020-05-11

**Authors:** Dragos Constantin Popescu, Ioan Dumitrache, Simona Iuliana Caramihai, Mihail Octavian Cernaianu

**Affiliations:** 1 Faculty of Automatic Control and Computers, University Politehnica of Bucharest, Splaiul Independentei No. 313, 060042 Bucharest, Romania; ioan.dumitrache@acse.pub.ro (I.D.); simona.caramihai@aii.pub.ro (S.I.C.); 2 Extreme Light Infrastructure-Nuclear Physics (ELI-NP)/Horia Hulubei National Institute for R&D in Physics and Nuclear Engineering (IFIN-HH), Str. Reactorului No. 30, Magurele, 077125 Bucharest, Romania; 3 Department of Science and Information Technology of the Romanian Academy, Cal. Victoriei No. 125, 010071 Bucharest, Romania

**Keywords:** critical infrastructures, positioning system, optical measurements, fiducial markers, adaptive kalman, measurement fusion

## Abstract

The paper addresses the problem of fusing the measurements from multiple cameras in order to estimate the position of fiducial markers. The objectives are to increase the precision and to extend the working area of the system. The proposed fusion method employs an adaptive Kalman algorithm which is used for calibrating the setup of cameras as well as for estimating the pose of the marker. Special measures are taken in order to mitigate the effect of the measurement noise. The proposed method is further tested in different scenarios using a Monte Carlo simulation, whose qualitative precision results are determined and compared. The solution is designed for specific positioning and alignment tasks in physics experiments, but also, has a degree of generality that makes it suitable for a wider range of applications.

## 1. Introduction

The evolution of technology has led to an increasing demand for solving complex problems which may be viewed as attempts to control and direct system behaviours towards desired states. The inherent complexity of problems and processes requires new approaches both in system modelling and in defining the emergent interaction with a highly dynamical and sparsely defined environment.

Cognitive approaches are successfully used in contexts where the boundary between the systems and the environment is fuzzy. However, they exhibit strong interrelation and interconnection, assisted by specific perception mechanisms [1]. Advances in complex control applications can be achieved only by considering adequate design approaches for sensory systems, especially in domains like environmental applications [2], health [3], industrial control, agriculture, etc.

Although new technologies such as wireless sensor networks or Internet of Things (IoT) are providing valuable solutions for appropriate perception mechanisms, complex issues are raised with the inclusion of data fusion, reliability, flexibility, reconfigurability, and cost of the measurement system. If some of these problems can be addressed during the modelling process, there are others (e.g., sensor positioning and sensor dynamics) that have a bigger impact on the generality of the overall solution.

These issues are specific in critical infrastructures, research institutes, power systems, e-health, mining, and traffic control, where the multitude of concurrent dynamics generating a large amount of information requires adequate solving methods that cover aspects such as relevance, efficiency, and cost optimisation.

This paper follows the development of a measurement method used in a very precise and yet flexible and portable positioning system. Firstly, the method is an answer to a specific application: Automatic alignment with high precision of various instruments which have to be remotely operated in highly flexible, open, and dynamic configurations for physics experiments. However, it can be used in any application where high precision positioning over a large working area is required, where no absolute reference can be defined, or when it is necessary to simultaneously align multiple features in relative positions, with high reliability. Such applications include large scale construction sites where multiple components need to be positioned in precise locations, manufacturing facilities where large machines are assembled or spacecraft docking in zero gravity environments [4].

The article has the following structure. The specific application and context addressed in this work are presented in Section 2. The theoretical context behind the proposed algorithm is described in Section 3. It it then used to build two essential procedures for the positioning system. The precision of the proposed method is assessed in the Section 4 using a Monte Carlo simulation. The conclusions and future developments are detailed in Section 5.

## 2. Description of the Initial Problem

The large number of high repetition rate, ultrashort pulse, and high power laser facilities that will come online all around the world will require state-of-the-art tools to allow the harnessing of their full potential [5]. The high power lasers of the ELI-NP (Extreme Light Infrastructure-Nuclear Physics) user facility will be employed for a wide range of research topics like studies of nuclear induced reactions, dark matter search, material irradiation, or medical applications [6]. The development of the experimental setups for studying these topics requires specific instrumentation, while also having strict needs in terms of positioning and alignment, in order to ensure optimal experimental conditions.

**Figure 1:**
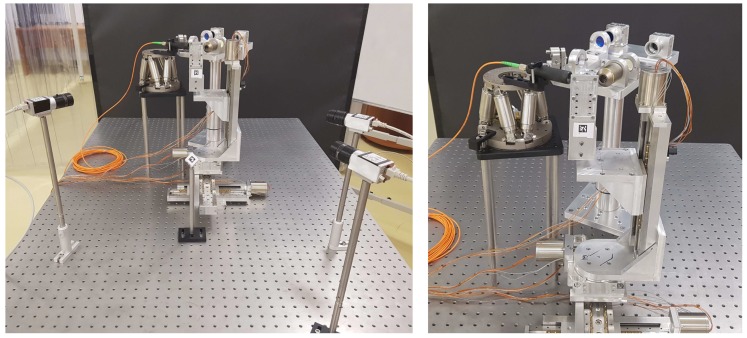
Solid target alignment setup example where multiple instruments are positioned using motorised manipulators.

As the setups are continuously changing, absolute position referencing is hard to achieve. This is a necessity, as multiple instruments need to be precisely positioned relative to each other during the experiment. Figure 1 displays an example of a setup for solid target alignment in which multiple optical diagnostics are positioned using motorised manipulators that have 3 to 6 degrees of freedom (DOF).

Apart from the precision requirements, additional ones should be taken into consideration. The alignment should be done remotely because the experiments take place inside vacuum chambers and behind concrete walls used for radiation shielding. Moreover, to take advantage of the high repetition rate of the lasers and to maximise the beam-time available for the users, the positioning has to be performed in a limited amount time. In a previous work [7], we addressed this series of requirements and constraints by developing an automatic alignment system that is based on relative position measurements using imaging cameras and compact and flat fiducial markers, due to space and visibility constraints. The alignment algorithm was based on a real-time optimisation procedure which is the subject of a patent [8].

Although the fiducial markers were initially developed and used in augmented reality applications [9], due to their versatility in determining their position in a non-invasive manner (by imaging them with a camera), they were quickly adopted for a different range of applications. These include kinematic calibration and visual servoing for industrial robots [10,11], robot localisation and navigation [12,13,14], SLAM (simultaneous localisation and mapping) [15,16,17] and sensor fusion [18,19,20].

The motivation behind this work is to combine the measurements from multiple cameras in order to increase the working area of the system and to maximise the positioning precision. Simultaneously, the main focus is to present and test the precision of the proposed methods and algorithm, comparing the results with those recorded while using only one camera [21].

## 3. Method

The basic approach behind the proposed method is to use multiple cameras in order to improve the precision of estimating the pose of fiducial markers and to extend the working area of the system. It begins with the assumption that each camera provides measurements that are erroneous and noisy. The problem can be conceived through an analogy with the GPS system where distance measurements from multiple satellites are used to estimate the position of the receiver module with high precision. The key ingredient in the GPS system is to know very precisely the position of the satellites. The proposed positioning system needs to meet the same requirement for its cameras, but wasting too much effort on this task diminishes its practicality. Consequently it is only assumed that the cameras have unknown but fixed positions.

A related approach that has similar objectives can be found in [22]. The main benefit of the method is the possibility to calibrate the setup of cameras using a 3D feature with fiducial markers having unknown configuration. However, it is not meant for estimating the pose of single markers. In order to achieve this, additional methods are required.

Our method is developed using the ArUco fiducial markers [23,24]. The ArUco library detects and estimates the pose of the marker with respect to the camera using the solvepnp algorithm in which the pinhole camera model is data fitted using a Levenberg–Marquardt non-linear optimisation procedure. The output is represented by the extrinsic camera parameters which can be expressed in terms of a homogenous transformation $T_{C}^{M}$ (the transformation between the camera attached reference frame and the marker attached one) detailed in [7].

### 3.1. Adaptive Kalman Fusion Algorithm for Multiple Cameras and Fiducial Markers

The method consists of fusing noisy measurements from multiple cameras, in order to improve the precision of estimating the exact position of the fiducial marker. In the scientific literature, multiple sensor fusion and noise filtering methods have been developed over the years. Among them there is the Kalman filter [25], which is the most widely used. The noise effect is mitigated by using a dynamical model for the physical process involved and a statistical model (covariance matrix) for the noise in the measurement process.

For a discrete linear state-space dynamical model in Equation (1), the Kalman filter estimates the value of the state vector *x* using noisy measurements for the input *u* and the output *y*.

(1) $$\left\{\begin{array}{c} x_{k}=A\cdot x_{k-1}+B\cdot u_{k-1}+q_{k-1}\\ y_{k}=C\cdot x_{k}+r_{k} \end{array}\right..$$

In standard data fusion applications that use the Kalman filter, the pose of various objects is estimated using the input from accelerometer, gyroscope, and magnetometer sensors [26,27] and the output from distance measurement devices [28,29]. Our application makes impossible to use any type of attached sensors and, hence, we only rely on the “non-invasive” pose measurement using the solvepnp algorithm and imaging cameras.

Our approach is to employ a state-space model with free dynamics (where *u* is zero) and with an identity state matrix (*A*). In this respect, each measurement is made using only one snapshot image from all the cameras that are synchronised with the help of an electrical trigger signal. In this way, the position of the fiducial marker during the measurement is considered fixed. On the same set of images, the solvepnp algorithm is applied multiple times and, thus, the evolution of the measurement is caused only by the noise and not by the movement of the fiducial marker. Furthermore, the measurements are used to iteratively estimate the real position using the proposed algorithm.

In order to improve the results, we propose a procedure designed to update the statistical model of the noise. The *Q* and *R* covariance matrices (which are discussed below) are continuously adjusted using the newly acquired data. Any change of the noise behaviour and any existing correlations are captured and thus, the effect of the noise is mitigated more efficiently. Consequently, the proposed algorithm is an adaptive Kalman version.

The homogeneous transformation representation ($T_{C}^{M}$) has numerous practical benefits, especially for pose composition and inversion operations, but it is not suitable in this circumstance because it is redundant (16 numerical values for expressing a 6 DOF pose). The solution is to use an equivalent representation which is composed of the set of translation coordinates (*X*, *Y*, and *Z*) and the set of Euler angles (AX, AY, and AZ).

In this particular case, the structure of the state vector is the real pose of the fiducial marker defined in Equation (2), where the subscript *k* denotes the present discrete-time sample of the state vector.

(2) $$x_{k}=\left[\begin{array}{c} X_{k}\\ Y_{k}\\ Z_{k}\\ AX_{k}\\ AY_{k}\\ AZ_{k} \end{array}\right].$$

The output vector is composed of the pose elements measured using all the cameras available. The structure of the output vector is defined in Equation (3), where the superscript *i* is indicating the index of the camera considered.

(3) $$y_{k}=\left\lceil \begin{array}{c} X_{k}^{1}\\ Y_{k}^{1}\\ Z_{k}^{1}\\ AX_{k}^{1}\\ AY_{k}^{1}\\ AZ_{k}^{1}\\ \vdots \end{array}\right\rceil \;\;\;\left\lfloor \begin{array}{c} \vdots \\ X_{k}^{i}\\ Y_{k}^{i}\\ Z_{k}^{i}\\ AX_{k}^{i}\\ AY_{k}^{i}\\ AZ_{k}^{i}\\ \vdots \end{array}\right\rfloor .$$

The discrete-time state-space model considered is defined in Equation (4), where $I_{6}$ is the identity matrix of rank 6, $q_{k-1}$ is a random noise signal with normal distribution (white noise) that is quantifying the false evolution induced by the noisy measurement, the *H* is the measurement model matrix defined in Equation (5), and $r_{k}$ is the measurement noise also considered white noise.

(4) $$\left\{\begin{array}{c} x_{k}=I_{6}\cdot x_{k-1}+q_{k-1}\\ y_{k}=H\cdot x_{k}+r_{k} \end{array}\right.$$

(5) $$H=\left[\begin{array}{c} I_{6}^{1}\\ \vdots \\ I_{6}^{i}\\ \vdots \end{array}\right].$$

The equations that describe the Kalman filter are presented in Equation (6). The first two equations give a rough state estimate using the dynamical model while the last 5 equations are used for improving the estimate using the newly acquired output sample. *P* is a covariance matrix that expresses the confidence degree of the state estimation, which is updated during the algorithm iterations, *Q* and *R* are the covariance matrices associated with the noises *q* and *r* respectively, $v_{k}$ is the rough estimation error (difference between the measured output and the predicted one using the first two equations), $S_{k}$ is the covariance matrix associated with the predicted output, and $K_{k}$ is the Kalman state update.

(6) $$\begin{array}{c} \mathrm{Predict}\mathrm{the}\mathrm{state}\\ 1.\;{\hat{x}}_{k|k-1}=A\cdot {\hat{x}}_{k-1|k-1}\\ 2.\;P_{k|k-1}=A\cdot P_{k-1|k-1}\cdot A^{T}+Q_{k-1}\\ \mathrm{Update}\mathrm{the}\mathrm{prediction}\\ 3.\;v_{k}=y_{k}-H_{k}\cdot {\hat{x}}_{k|k-1}\\ 4.\;S_{k}=H_{k}\cdot P_{k|k-1}\cdot H_{k}^{T}+R_{k}\\ 5.\;K_{k}=P_{k|k-1}\cdot H_{k}^{T}\cdot S_{k}^{-1}\\ 6.\;{\hat{x}}_{k|k}={\hat{x}}_{k|k-1}+K_{k}\cdot v_{k}\\ 7.\;P_{k|k}=P_{k|k-1}-K_{k}\cdot S_{k}\cdot K_{k}^{T} \end{array}$$

The filter requires good estimates for the initial state $x_{0}$ and the covariance matrices *Q* and *R*. In the proposed algorithm this is achieved using an initialisation procedure. The position of the fiducial marker is measured for $N_{i}$ number of sampling times. The result is the set of samples defined in Equation (3) for $k=1,\cdots ,N_{i}$ (the length of the initialisation) and i=1,⋯,n (the number of cameras available).

Averaging the samples gives a good estimate for the initial state, which is built according to Equation (7), where E[·] is the mean operator (expected value). The *Q* and *R* matrices are computed from the same set of samples, assuming that there is no correlation between the noises affecting the measurements.

(7) $$x_{0}=\left[\begin{array}{c} X_{0}\\ Y_{0}\\ Z_{0}\\ AX_{0}\\ AY_{0}\\ AZ_{0} \end{array}\right]=\left[\begin{array}{c} \underset{\begin{array}{c} 1\leq k\leq N_{i}\\ 1\leq i\leq n \end{array}}{E}\left[X_{k}^{i}\right]\\ \underset{\begin{array}{c} 1\leq k\leq N_{i}\\ 1\leq i\leq n \end{array}}{E}\left[Y_{k}^{i}\right]\\ \underset{\begin{array}{c} 1\leq k\leq N_{i}\\ 1\leq i\leq n \end{array}}{E}\left[Z_{k}^{i}\right]\\ \underset{\begin{array}{c} 1\leq k\leq N_{i}\\ 1\leq i\leq n \end{array}}{E}\left[AX_{k}^{i}\right]\\ \underset{\begin{array}{c} 1\leq k\leq N_{i}\\ 1\leq i\leq n \end{array}}{E}\left[AY_{k}^{i}\right]\\ \underset{\begin{array}{c} 1\leq k\leq N_{i}\\ 1\leq i\leq n \end{array}}{E}\left[AZ_{k}^{i}\right] \end{array}\right]$$

(8) $$\begin{array}{c} Q_{0}=diag(\underset{\begin{array}{c} 1\leq i\leq n \end{array}}{E}\left[\underset{\begin{array}{c} 1\leq k\leq N_{i} \end{array}}{var}\left(X_{k}^{i}\right)\right],\underset{\begin{array}{c} 1\leq i\leq n \end{array}}{E}\left[\underset{\begin{array}{c} 1\leq k\leq N_{i} \end{array}}{var}\left(Y_{k}^{i}\right)\right],\underset{\begin{array}{c} 1\leq i\leq n \end{array}}{E}\left[\underset{\begin{array}{c} 1\leq k\leq N_{i} \end{array}}{var}\left(Z_{k}^{i}\right)\right],\\ \underset{\begin{array}{c} 1\leq i\leq n \end{array}}{E}\left[\underset{\begin{array}{c} 1\leq k\leq N_{i} \end{array}}{var}\left(AX_{k}^{i}\right)\right],\underset{\begin{array}{c} 1\leq i\leq n \end{array}}{E}\left[\underset{\begin{array}{c} 1\leq k\leq N_{i} \end{array}}{var}\left(AY_{k}^{i}\right)\right],\underset{\begin{array}{c} 1\leq i\leq n \end{array}}{E}\left[\underset{\begin{array}{c} 1\leq k\leq N_{i} \end{array}}{var}\left(AZ_{k}^{i}\right)\right]) \end{array}$$

(9) $$R_{0}=diag\left(R_{0}^{1},\cdots ,R_{0}^{i},\cdots ,R_{0}^{n}\right)$$

(10) $$\begin{array}{c} R_{0}^{i}=diag(\underset{\begin{array}{c} 1\leq k\leq N_{i} \end{array}}{var}\left(X_{k}^{i}\right),\underset{\begin{array}{c} 1\leq k\leq N_{i} \end{array}}{var}\left(Y_{k}^{i}\right),\underset{\begin{array}{c} 1\leq k\leq N_{i} \end{array}}{var}\left(Z_{k}^{i}\right),\\ \underset{\begin{array}{c} 1\leq k\leq N_{i} \end{array}}{var}\left(AX_{k}^{i}\right),\underset{\begin{array}{c} 1\leq k\leq N_{i} \end{array}}{var}\left(AY_{k}^{i}\right),\underset{\begin{array}{c} 1\leq k\leq N_{i} \end{array}}{var}\left(AZ_{k}^{i}\right)). \end{array}$$

*Q* is a diagonal matrix defined in Equation (8) built using the mean variance of the pose elements (*X*, *Y*, *Z*, AX, AY, and AZ) along all the cameras. The *R* matrix is defined in Equation (9) where $R^{i}$ is defined in Equation (10), built using the variance for each pose element measured by each camera.

After the initialisation is finished, the Kalman algorithm is iterated for $N_{e}$ number of sampling times, while at each step, a new set of samples $y_{k}$ in the form of Equation (3) is measured and an estimated state trajectory is built (${\hat{x}}_{k|k}$ for $k=N_{i}+1,\cdots ,N_{i}+N_{e}$).

Newly measured system outputs can be used to improve the statistical models of the noises for increased performance. Thereby, at each iteration, every new set is added to the initialisation set and the covariance matrices *Q* and *R* are updated using Equations (11)–(13) for $k=1,\cdots ,N_{e}$.

(11) $$\begin{array}{c} Q_{k}=diag(\underset{\begin{array}{c} 1\leq i\leq n \end{array}}{E}\left[\underset{\begin{array}{c} 1\leq j\leq N_{i}+k \end{array}}{var}\left(X_{j}^{i}\right)\right],\underset{\begin{array}{c} 1\leq i\leq n \end{array}}{E}\left[\underset{\begin{array}{c} 1\leq j\leq N_{i}+k \end{array}}{var}\left(Y_{j}^{i}\right)\right],\underset{\begin{array}{c} 1\leq i\leq n \end{array}}{E}\left[\underset{\begin{array}{c} 1\leq j\leq N_{i}+k \end{array}}{var}\left(Z_{j}^{i}\right)\right],\\ \underset{\begin{array}{c} 1\leq i\leq n \end{array}}{E}\left[\underset{\begin{array}{c} 1\leq j\leq N_{i}+k \end{array}}{var}\left(AX_{j}^{i}\right)\right],\underset{\begin{array}{c} 1\leq i\leq n \end{array}}{E}\left[\underset{\begin{array}{c} 1\leq j\leq N_{i}+k \end{array}}{var}\left(AY_{j}^{i}\right)\right],\underset{\begin{array}{c} 1\leq i\leq n \end{array}}{E}\left[\underset{\begin{array}{c} 1\leq j\leq N_{i}+k \end{array}}{var}\left(AZ_{j}^{i}\right)\right]) \end{array}$$

(12) $$R_{k}=diag\left(R_{k}^{1},\cdots ,R_{k}^{i},\cdots ,R_{k}^{n}\right)$$

(13) $$\begin{array}{c} R_{k}^{i}=diag(\underset{\begin{array}{c} 1\leq j\leq N_{i}+k \end{array}}{var}\left(X_{j}^{i}\right),\underset{\begin{array}{c} 1\leq j\leq N_{i}+k \end{array}}{var}\left(Y_{j}^{i}\right),\underset{\begin{array}{c} 1\leq j\leq N_{i}+k \end{array}}{var}\left(Z_{j}^{i}\right),\\ \underset{\begin{array}{c} 1\leq j\leq N_{i}+k \end{array}}{var}\left(AX_{j}^{i}\right),\underset{\begin{array}{c} 1\leq j\leq N_{i}+k \end{array}}{var}\left(AY_{j}^{i}\right),\underset{\begin{array}{c} 1\leq j\leq N_{i}+k \end{array}}{var}\left(AZ_{j}^{i}\right)). \end{array}$$

**Figure 2:**
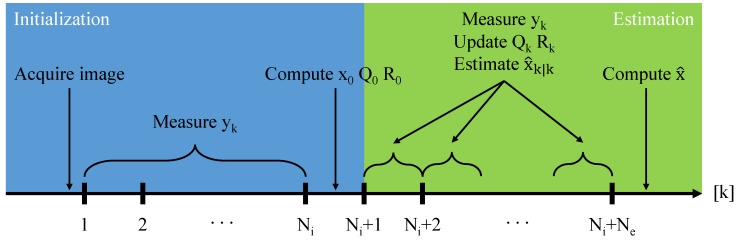
The sequence of procedures involved in the proposed algorithm.

Considering that the estimated state trajectory (${\hat{x}}_{k|k}$) belongs to a system with free dynamics where, in the absence of the noise effects, the state should be constant, a final estimate with a better precision can be achieved by averaging the values of the estimated state trajectory according to Equation (14). The timeline of all the procedures that are involved in the proposed algorithm is presented in Figure 2. In Algorithm 1 the proposed procedure is summarised.

(14) $$\hat{x}=\underset{N_{i}+1\leq k\leq N_{i}+N_{e}}{E}\left[{\hat{x}}_{k|k}\right]$$

**Algorithm 1:** The proposed algorithm for multi-camera pose fusion.
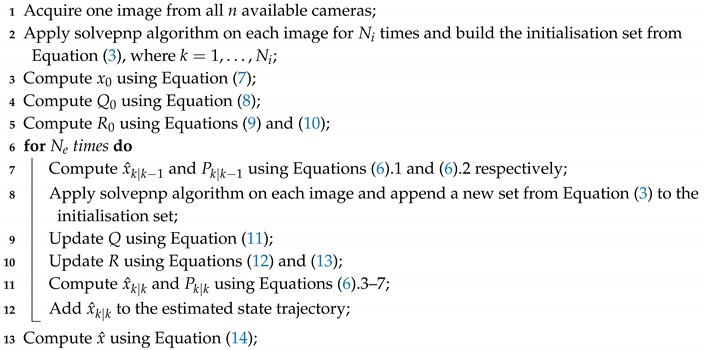


### 3.2. Setup Calibration Procedure

**Figure 3:**
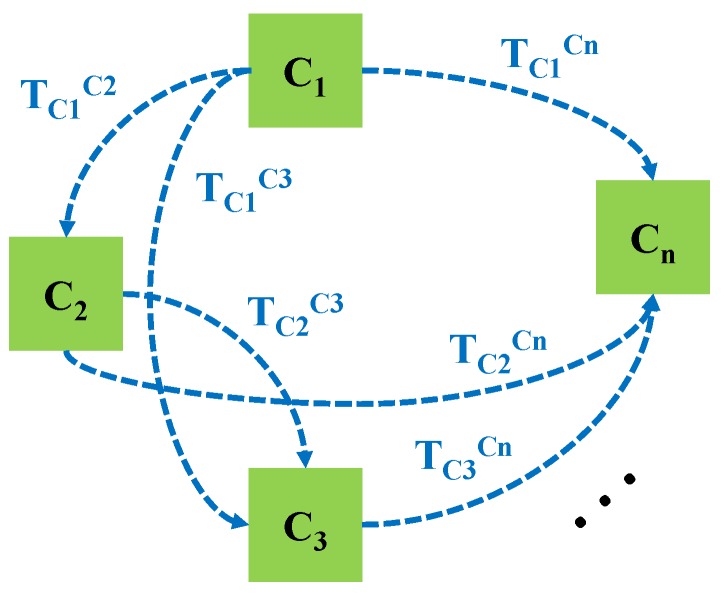
The calibration of a set of *n* cameras requires to determine their relative positions $T_{Ci}^{Cj}$.

Given a set of *n* cameras in a pre-defined fixed configuration, having unknown absolute positions, the purpose of the setup calibration procedure is to determine, in a precise manner, their relative positions. As depicted in Figure 3, the goal is to determine the set of homogenous transformations $T_{Ci}^{Cj}$ where i=1,⋯,n and j=i+1,⋯,n.

**Figure 4:**
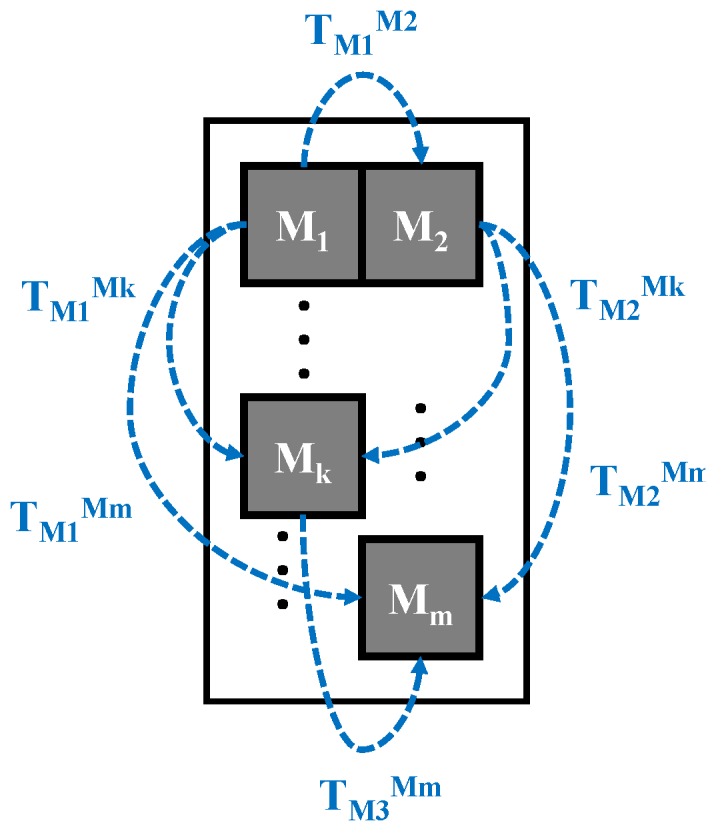
A setup of cameras is calibrated using a precision gauge. It is a manufactured feature having *m* fiducial markers where all their relative positions are precisely known.

This requires the use of a precision gauge, which can be manufactured in the form of a cube of fiducial markers like in [21] or in any 3D shape where the markers can be viewed all around. The gauge needs to be manufactured or calibrated with increased precision. It can be seen as the absolute precision reference used to calibrate the entire positioning system. In Figure 4, a conceptual diagram of a gauge containing *m* fiducial markers is presented. The set of homogenous transformations between all the markers ($T_{Mi}^{Mj}$ where i=1,⋯,m and j=i+1,⋯,m) is precisely known.

**Figure 5:**
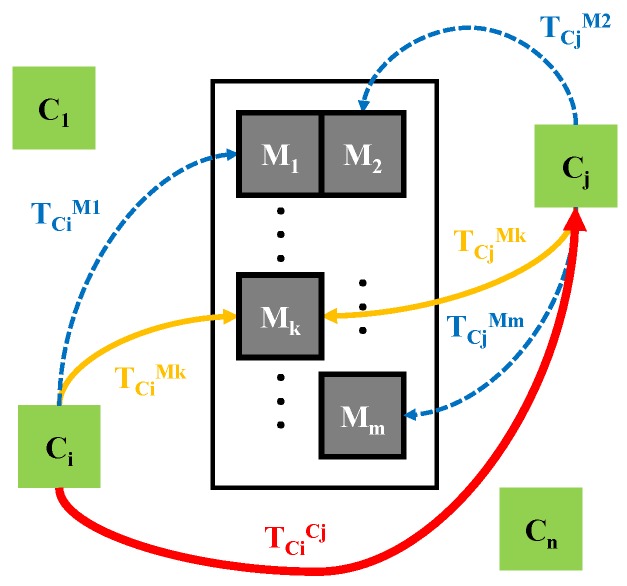
The process of calibrating all the pairs of *n* cameras, which illustrates all the homogenous transformations involved in finding the relative position of $C_{i}$ and $C_{j}$ cameras.

The camera setup calibration procedure is done simultaneously for all the camera pairs $C_{i}$, $C_{j}$ where i=1,⋯,n and j=i+1,⋯,n, with the goal of estimating the homogenous transformation $T_{Ci}^{Cj}$ (as presented in Figure 5). It starts with placing the precision gauge inside the environment. Depending on the orientation, each camera sees a different portion of the gauge, i.e., from all *n* fiducial markers, $C_{i}$ can measure the position of $n_{i}$ markers and $C_{j}$ can measure only $n_{j}$, where $n_{i}\leq n$ and $n_{j}\leq n$. It is preferable that at least one fiducial marker $M_{k}$ is seen by both cameras, otherwise, it can be virtually determined using the gauge transformations.

The proposed algorithm estimates the $T_{Ci}^{Mk}$ homogenous transformation using the noisy measurements from all $n_{i}$ markers using the $C_{i}$ camera. This transformation is expressed multiple times in terms of $T_{Ci}^{Ml}$ pose measurement (where $l=1,\cdots ,n_{i}$) using Equation (15). The $T_{Ml}^{Mk}$ transformation is precisely known from the gauge.

(15) $$T_{Ci}^{Mk}\approx {\hat{T_{Ci}^{Mk}}}_{l}=T_{Ci}^{Ml}\cdot T_{Ml}^{Mk}$$

The resulting ${\hat{T_{Ci}^{Mk}}}_{l}$ pose is converted to translations and Euler angles which are used for building the output measurement vector of Equation (3).

The proposed algorithm is further applied and the result is an estimation of $T_{Ci}^{Mk}$ having a higher degree of precision and being closer to the real value. For the $C_{j}$ camera, $T_{Cj}^{Mk}$ is estimated in a similar manner. Consequently, $T_{Ci}^{Cj}$ transformation is computed using Equation (16).

(16) $$T_{Ci}^{Cj}=T_{Ci}^{Mk}\cdot {\left(T_{Mk}^{Cj}\right)}^{-1}$$

### 3.3. Position Estimation Procedure

Having a calibrated camera setup, the aim of this procedure is to estimate the position of a fiducial marker attached to a specific instrument, according to the application.

**Figure 6:**
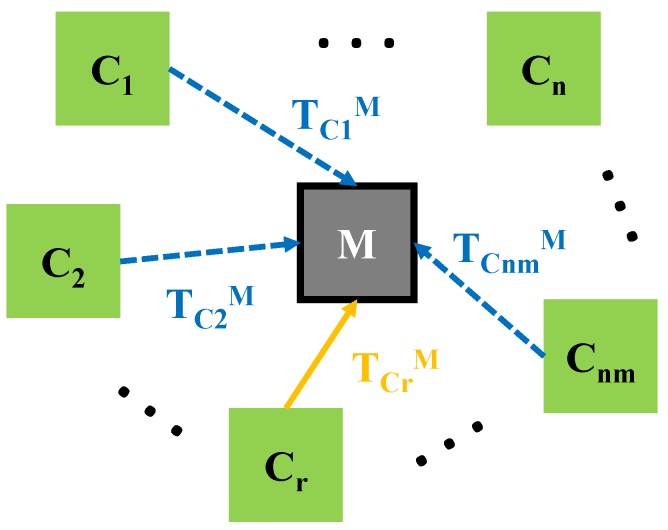
The homogenous transformations involved in estimating the position of a fiducial marker placed in the environment of a calibrated camera setup.

In Figure 6, the conceptual diagram of this procedure is depicted. Depending on the configuration, the *M* marker can be seen only by a number of $n_{m}$ out of *n* cameras ($n_{m}\leq n$). An arbitrary camera ($C_{r}$) is chosen, which is considered the positioning reference.

The proposed algorithm is estimating the $T_{Cr}^{M}$ homogenous transformation using the noisy measurements from each of the $n_{m}$ cameras. In a similar manner to the setup calibration procedure, the $T_{Cr}^{M}$ transformation is expressed multiple times in terms of $T_{Ck}^{M}$ pose measurement (where $k=1,\cdots ,n_{m}$) using Equation (17). The $T_{Cr}^{Ck}$ is precisely known from the setup calibration.

(17) $$T_{Cr}^{M}\approx {\hat{T_{Cr}^{M}}}_{k}=T_{Cr}^{Ck}\cdot T_{Ck}^{M}.$$

The resulting ${\hat{T_{Cr}^{M}}}_{k}$ pose is converted to translations and Euler angles and used for building the output measurement vector of Equation (3). The proposed algorithm is further applied and the result is an estimation of $T_{Cr}^{M}$ which has a higher degree of precision and is closer to the real value.

This approach can be used in a similar manner for measuring the relative position of multiple fiducial markers in the environment, which is very useful in alignment tasks as presented in [7].

## 4. Simulation Results

### 4.1. Monte Carlo Setup

There are two factors that contribute and affect the precision of estimating the pose of the fiducial marker. First, there are the physical and environment-related aspects, which include the optical specifications of the imaging system (the sensor and optical resolution, the focal length, the depth of field, and the field of view), the environment illumination conditions (how strong, uniform, and consistent the lighting is) and if the cameras are optimally positioned so as to achieve good viewing angles and maintain consistent accuracy along the working area. Secondly, there are factors related to the algorithms regarding how precise the pose can be estimated and how much the effect of the noise can be mitigated during the data fusion. In this paper we decouple the two types of factors and only consider the behaviour of our proposed method, so as to have a first qualitative assessment regarding the precision.

Consequently, we developed a simulation environment that aims to replicate how our method performs in a real setup, considering the noisy pose estimations it receives from the solvepnp algorithm. For an increased confidence in the results, we adopted a Monte Carlo approach in which we statistically analysed how the noise is propagated through our method and how the precision is affected. The simulation is implemented in MATLAB where positions of multiple cameras, precision gauges, and fiducial markers are virtually defined. In order to simulate the noisy input from the solvepnp, each position (that is supposed to be measured in the real environment) is disturbed with additive random noise having normal distribution. The noise is configured considering the precision limits we determined experimentally for one camera in our previous work [21]: for *X* and *Y*, 75μm, for *Z*, 300μm, and for AX, AY and AZ, $0.02^{\circ }$. The mean value of the noise is 0 while the standard deviation (σ) was configured in such a way that 95.45% of the noise values are within the experimental limits (inside [−2σ,2σ]).

The simulation puts in place three scenarios which are additionally used to assess the different contributions between the number of cameras and the number of the fiducial markers from the gauge:

1. 3 cameras, a precision gauge with 5 markers, and one marker whose position must be estimated;
2. 5 cameras, a precision gauge with 3 markers, and one marker whose position must be estimated;
3. 5 cameras, a precision gauge with 5 markers, and one marker whose position must be estimated.

The results are compared against estimating the pose using only one camera in the same environment. The Monte Carlo simulation performs 5000 iterations where, in each run and for each of the scenarios, the setup is calibrated using the gauge. The calibration is further used to estimate the pose of the fiducial marker which is compared with the true, predefined one. The $N_{i}$ and $N_{e}$ parameters are configured to 20 and 50 respectively. In a real application, the choice of these two parameters is a matter of cost optimisation, considering the computational effort, the resources available, and the required measuring frequency.

Compared with a real setup, we adopted one simplifying hypothesis. The angle of incidence of the marker relative to the camera, as we showed in [21], has an influence on the precision. Close to normal angles of incidence tend to bring more noise in the estimation. In this study, we only consider that the precision of the solvepnp algorithm to be consistent, regardless of the angle. However, in other respects, the simulation is considering the worst case scenario because of the following arguments:

- Noises from different cameras and from different elements of the pose (*X*, *Y*, *Z*, AX, AY, and AZ) are considered completely uncorrelated. In real circumstances this might not be the case (e.g., the noise induced by the environment illumination which affects all the measurements in a similar manner) thus, any relaxed conditions are contributing to an increased precision of the estimation. This additional information is harnessed using the update procedure for *Q* and *R* covariance matrices (which in this case would no longer be diagonal);
- The precision of one camera measurement along the *Z* axis is 5 times lower compared with the *X* and *Y* axis. In the simulation this is taken as it is, but in a real situation, this effect can be diminished by placing the camera setup in an optimal configuration. For instance, the measurement along the *Z* axis from one camera can be replaced by measurements from two cameras placed in lateral positions, for which the *Z* axis motion is decomposed in *X* and *Y* components that have a higher precision.

### 4.2. Results

**Figure 7:**
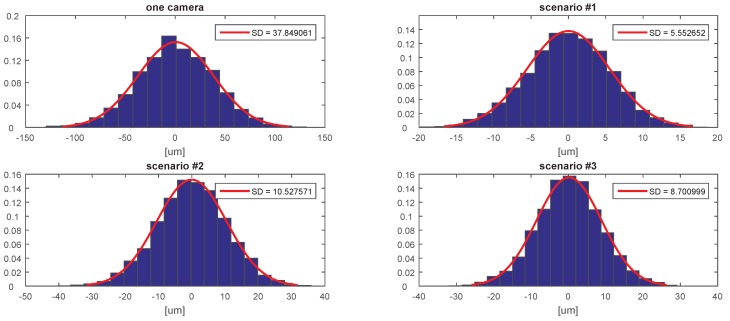
The probability density function (PDF) and the standard deviation (SD) of the error for estimating the *X* coordinate.

**Figure 8:**
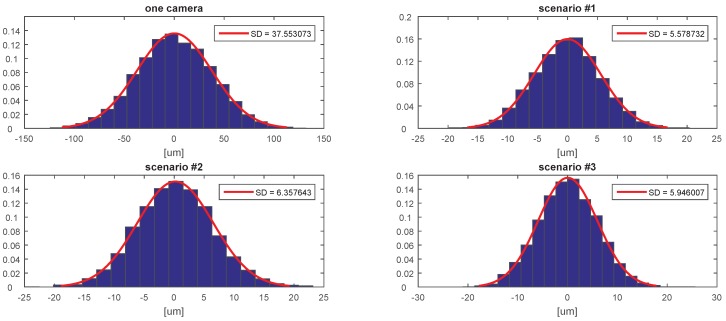
The probability density function (PDF) and the standard deviation (SD) of the error for estimating the *Y* coordinate.

**Figure 9:**
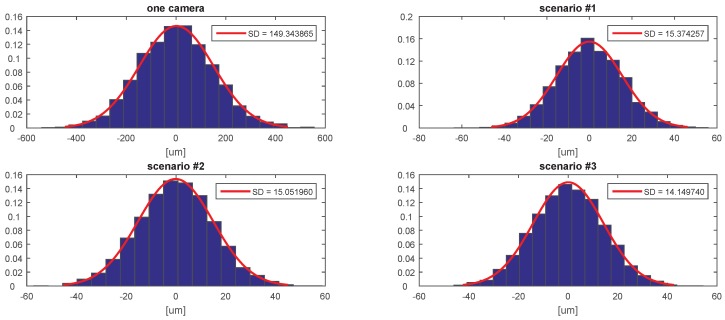
The probability density function (PDF) and the standard deviation (SD) of the error for estimating the *Z* coordinate.

**Figure 10:**
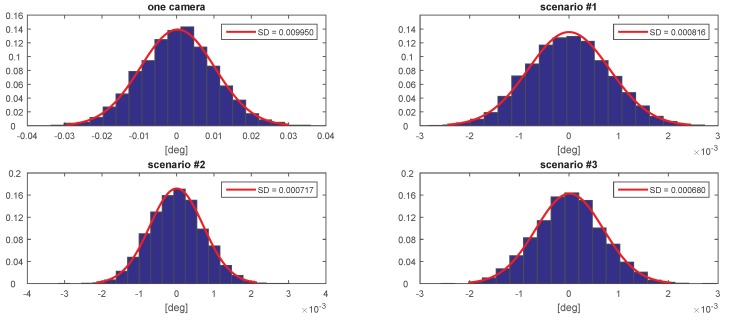
The probability density function (PDF) and the standard deviation (SD) of the error for estimating the AX angle.

**Figure 11:**
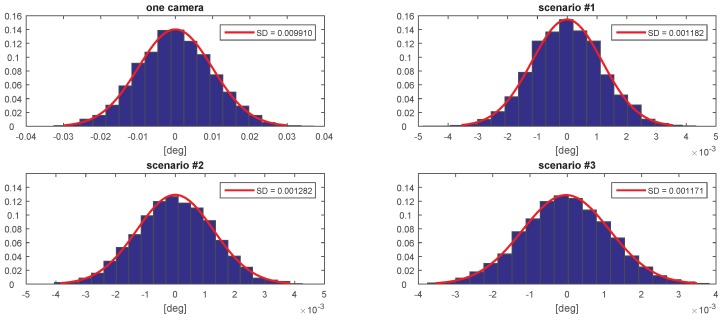
The probability density function (PDF) and the standard deviation (SD) of the error for estimating the AY angle.

**Figure 12:**
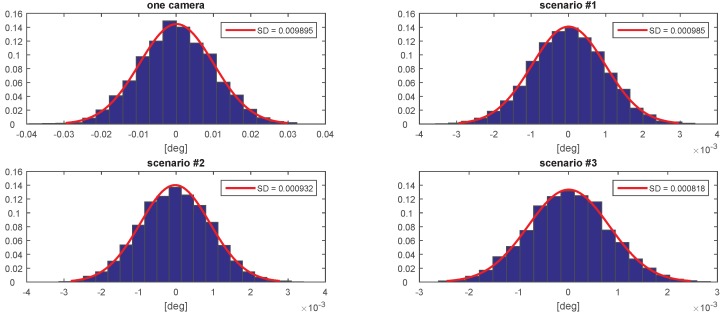
The probability density function (PDF) and the standard deviation (SD) of the error for estimating the AZ angle.

Figure 7, Figure 8, Figure 9, Figure 10, Figure 11 and Figure 12 presents the simulation results for estimating *X*, *Y*, and *Z* coordinates and AX, AY, and AZ orientation angles, which are given in terms of the probability density function (PDF) and the standard deviation (SD) of the error. The first plot from each Figure depicts the estimation error achieved using only one camera. The following three plots give the results achieved using the setups from each of the above-mentioned scenarios.

The results show that the proposed algorithm achieves an increase in precision which is close to an order of magnitude. It can also be observed that it is of greater importance to have more fiducial markers in the precision gauge instead of more cameras. In scenario #3, a slight decrease of precision is experienced in comparison to the #1 scenario. This is to be expected as each added camera is an additional noise source. However, the benefit of achieving a larger working area is far more important. In Table 1 the results are summarised and compared with regards to the limits of the variation interval [−2σ,2σ] where it is expected that 95.45% of the errors will occur. This can be considered the precision that the positioning system is expected to achieve when using th proposed method.

**Figure 13:**
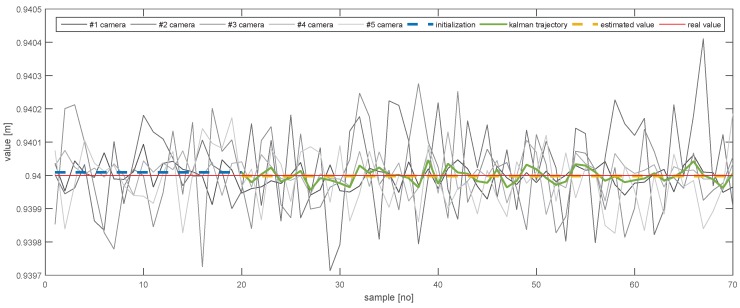
The evolution of the estimation of *X*.

**Figure 14:**
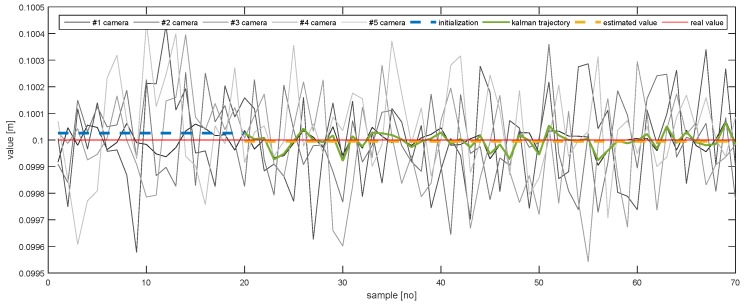
The evolution of the estimation of *Y*.

**Figure 15:**
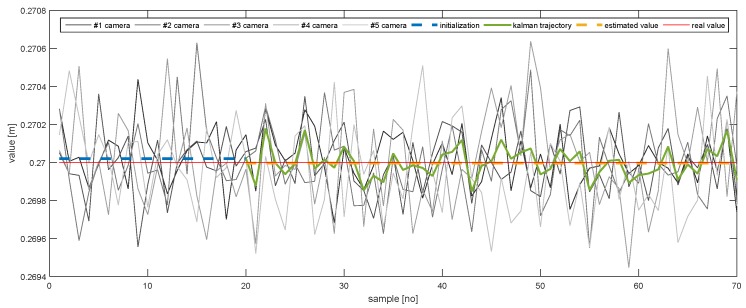
The evolution of the estimation of *Z*.

**Figure 16:**
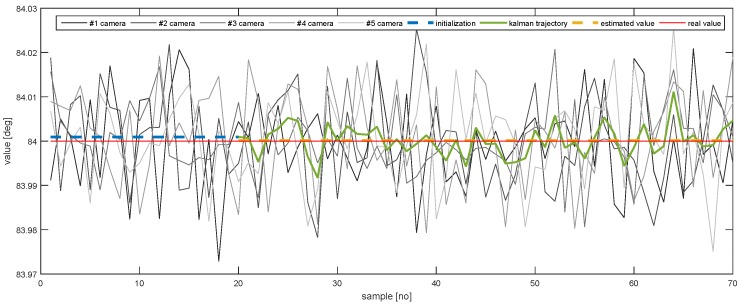
The evolution of the estimation of AX.

**Figure 17:**
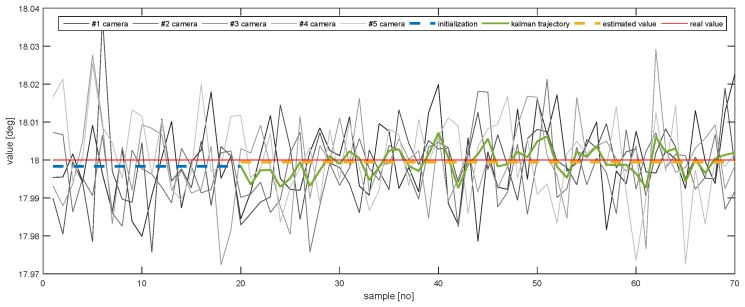
The evolution of the estimation of AY.

**Figure 18:**
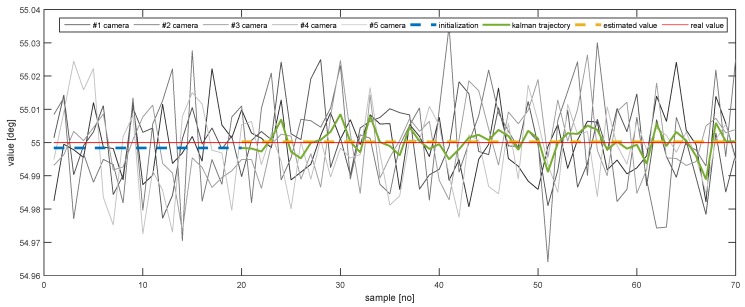
The evolution of the estimation of AZ.

In order to further emphasise the simulation results achieved by the proposed method, Figure 13, Figure 14, Figure 15, Figure 16, Figure 17 and Figure 18 depict a set of examples from the 3rd scenario which show how the estimation of the state vector elements is evolving. Each of the figures presents: The noisy measurement from the five cameras, the value of the state vector computed after the initialisation procedure, the evolution of the state vector during the Kalman estimation, and the final state estimation which falls close to the true value. Although the noise in the measurements is amplified because the reconstruction of the position of the marker in different cameras, the evolution of the Kalman estimation shows a clear noise damping effect.

## 5. Conclusions

With respect to the considered case study, the results show that the proposed method and algorithm have managed to successfully meet the objectives. The working area could be increased in accordance with the number of cameras in the setup. This is a decision-making procedure that needs to take into account the cost relative to the working area and the precision required. For our simulation scenarios, the precision increase was close to an order of magnitude, which was around 10–15 μm for *X* and *Y* coordinates, 30 μm for *Z* and $0.002^{\circ }$ for AX, AY, and AZ orientation angles.

The cost is an important benefit of the system compared to other solutions like laser trackers which tend to be extremely expensive. In addition to that, our proposed method could achieve relative and simultaneous positioning of multiple fiducial markers, which supports the development of advanced applications.

Future work will include a complete analysis of the method in a real environment where all physical and algorithm-related factors are considered, and a precision comparison against other methods presented in the literature.

## Figures and Tables

**Table 1:** The simulation results for estimating the pose using only one camera and multiple cameras in three scenarios. The value is the boundary of the error variation interval (2σ).

			Scenario		
Pose Element	UM	One Camera	#1	#2	#3
X	(μm)	75.68	11.1	21.04	17.4
Y	(μm)	75.1	11.14	12.7	11.88
Z	(μm)	298.68	30.74	30.01	28.28
AX	(deg)	0.019	0.0016	0.0014	0.0013
AY	(deg)	0.019	0.0022	0.0024	0.0022
AZ	(deg)	0.019	0.0019	0.0018	0.0016
